# Glibenclamide ameliorates the disrupted blood–brain barrier in experimental intracerebral hemorrhage by inhibiting the activation of NLRP3 inflammasome

**DOI:** 10.1002/brb3.1254

**Published:** 2019-03-11

**Authors:** Fulin Xu, Gang Shen, Zuopeng Su, Zijian He, Lutao Yuan

**Affiliations:** ^1^ Department of Neurosurgery Minhang District Central hospital Shanghai China

**Keywords:** BBB, glibenclamide, ICH, NLRP3

## Abstract

**Background:**

Glibenclamide is a widely used sulfonylurea drug prescribed to treat type II diabetes mellitus. Previous studies have demonstrated that glibenclamide has neuroprotective effects in central nervous system injury. However, the exact mechanism by which glibenclamide acts on the blood–brain barrier (BBB) after intracerebral hemorrhage (ICH) remains unclear. The purpose of this study was to validate the neuroprotective effects of glibenclamide on ICH and to explore the mechanisms underlying these effects.

**Methods:**

We investigated the effects of glibenclamide on experimental ICH using the autologous blood infusion model. Glibenclamide was administrated either immediately or 2 hr after ICH. Brain edema was quantified using the wet–dry method 3 days after injury. BBB integrity was evaluated by Evans Blue extravasation and degradation of the tight junction protein zona occludens‐1 (ZO‐1). mRNA levels of inflammatory cytokines were determined by quantitative polymerase chain reaction. Activation of the nucleotide‐binding oligomerization domain‐like receptor with a pyrin domain 3 (NLRP3) inflammasome and cell viability were also measured in cerebral microvascular endothelial b.End3 cells exposed to hemin. Neurological changes were evaluated by the Garcia score and rotarod test.

**Results:**

After ICH, the brain water content, Evans Blue extravasation, and inflammatory cytokines decreased significantly in the ipsilateral hemisphere of the experimental compared to the vehicle group. Glibenclamide treatment and NLRP3 knockdown significantly reduced hemin‐induced activation of the NLRP3 inflammasome, release of extracellular lactate dehydrogenase, apoptosis, and loss of ZO‐1 in b.End3 cells. However, NLRP3 knockdown abolished the protective effect of glibenclamide.

**Conclusion:**

Glibenclamide maintained BBB integrity in experimental ICH by inhibiting the activation of the NLRP3 inflammasome in microvessel endothelial cells. Our findings will contribute to elucidating the pharmacological mechanism of action of glibenclamide and to developing a novel therapy for clinical ICH.

## 1 INTRODUCTION

Intracerebral hemorrhage (ICH) is highly prevalent and detrimental to neurological functioning. Few effective treatment options are available for ICH, as the complex cellular response after ICH is unknown (Xi, Keep, & Hoff, 2006). Increasing evidence indicates that ICH triggers excessive inflammatory reactions due to the activation of immune cells and subsequent release of proinflammatory cytokines, which ultimately result in degradation of blood–brain barrier (BBB) and neuronal cell death (Ren et al., 2017). ICH‐induced disruption of the BBB further exacerbates the inflammatory response and disrupts BBB function (Keep et al., 2014). Therefore, modifying brain inflammation and maintaining BBB integrity are critical strategies for ICH therapy.

The ICH‐induced injurious inflammatory process involves a sterile inflammatory response triggered by tissue damage that is mediated through the nucleotide‐binding oligomerization domain‐like receptor with a pyrin domain 3 (NLRP3) inflammasome. The NLRP3 inflammasome is involved in caspase‐1‐mediated activation and release of the cytokines interleukin (IL)‐1β and IL‐18, which crucially contribute to ICH injury and post‐ICH remodeling (Latz, Xiao, & Stutz, 2013). Recent evidence demonstrates that upregulation of NLRP3 significantly amplifies neuroinflammation and increases brain edema after ICH (Ma et al., 2014; Zheng, Chen, Zhang, & Hu, 2016). Thus, blocking the activation of NLRP3 inflammasome may effectively reduce the severity of ICH injury.

Glibenclamide is a widely used sulfonylurea drug employed to treat type II diabetes mellitus that reduces an excessive inflammatory response by inhibiting sulfonylurea receptor 1 (Sur1) and the regulatory subunit of the K_ATP_ channel following central nervous system (CNS) injury (Caffes, Kurland, Gerzanich, & Simard, 2015). Subarachnoid hemorrhage (SAH) increased Sur1‐Trpm4 channels expression in humans and rats. Sur1 inhibitor glibenclamide significantly attenuated neuroinflammation and improved cognitive function after SAH (Simard et al., 2009; Tosun et al., 2013). Additionally, glibenclamide was suggested to effectively inhibit inflammatory cells migration by inhibiting NLRP3 inflammasome assembly, thereby reducing inflammatory cells infiltration and preventing further organ damage in ischemic tissue (Gao et al., 2016; Satoh, Kambe, & Matsue, 2013). One study demonstrated that glibenclamide protects BBB integrity, which reduces the extravasated protein‐induced production of proinflammatory mediators and improves neurological outcomes after experimental ICH (Jiang et al., 2017). Therefore, we tested the hypothesis that glibenclamide attenuates the disruption of the BBB after ICH by inhibiting the activation of the NLRP3 inflammasome.

## 2 METHODS

### 2.1 Animals and the ICH model

All experimental protocols involving animals were approved by the Ethics Committee of Shanghai Minhang District Hospital, Shanghai, China. Adult male C57Bl/6 mice (20–25 g; Shanghai SLAC Laboratory Animal Corp.; Shanghai, China) were used for ICH mouse modeling by autologous blood injection as previously reported (Wang et al., 2015). Briefly, the mice were anesthetized and injected with 30 μl blood into the brain at a rate of 1 μl/min using a Hamilton syringe and a micro‐infusion pump (WPI; Sarasota, FL) attached to the stereotaxic device (Stoelting; Kiel, WI). When the animals were fully conscious, they were sent back to their home cage.

### 2.2 Drugs and chemicals

Glibenclamide (Sigma Aldrich, St. Louis, MO) dissolved in dimethyl sulfoxide (DMSO; Sigma Aldrich) was administered intraperitoneally at the dose of 10 μg right after ICH as previously reported (Xu, Yuan, Liu, Ding, & Tian, 2017). Glibenclamide dissolved in DMSO was diluted with culture medium to 10 μM. The dose was chosen based on a previous study and our experimental findings. Hemin (Beyotime; Nantong, China) was used for the cell viability assay, as the dose (60 μM) significantly increased the mortality rate of bEnd.3 cells.

### 2.3 Measurement of brain water content

Brain water content was quantified using the wet–dry method Wang et al., 2015). Briefly, bilateral hemisphere brain samples collected 3 days after ICH were immediately weighed to obtain wet weight, and then reweighed after dehydrated at 100°C for 24 hr to obtain dry weight. Brain water content was calculated according to the following formula: [(wet weight − dry weight)/wet weight] × 100%.

### 2.4 Behavioral testing

Neurological changes were assessed by a blinded observer at each time point post‐ICH, using the Garcia score and rotarod test (Zhong et al., 2013). The Garcia score is composed of six parameters: spontaneous activity, symmetry of movement (four limbs), symmetry of forelimbs, climbing, reaction to touch, and vibrissae touch. The rotarod test was used to evaluate motor deficits in mice pretrained for 3 days before ICH (Wang et al., 2015). The average time for mice to fall was recorded on days 1, 3, and 7 after ICH.

### 2.5 Cell cultures and cell viability assay

Mouse brain microvascular endothelial cells (bEnd.3) were purchased from the American Type Culture Collection (Manassas, VA) and cultured in DMSO (Gibco Laboratories) supplemented with 10% fetal bovine serum, 1% streptomycin, and 1% penicillin. Primary mouse brain cells were isolated and maintained as previously reported (Zhao et al., 2016). The bEnd.3 cells were pretreated with or without 10 μM glibenclamide for 30 min, then exposed to 60 μM hemin for 24 hr. Cytotoxicity was determined by measuring lactate dehydrogenase (LDH) activity in the culture medium.

### 2.6 sIRNA transfection

Small interfering RNA targeting NLRP3 (si‐NLRP3) or negative control siRNA (si‐NC) was transfected into bEnd.3 cells for 48 hr with the aid of Lipofectamine® 2000 transfection reagent (Invitrogen, Carlsbad, CA). Western blot was performed to validate the knockdown efficiency of NLPR3.

### 2.7 Flow cytometry analysis after Anexin V and PI staining

siRNA‐transfected or control bEnd.3 cells were pretreated with or without 10 μM glibenclamide for 30 min, then exposed to 60 μM hemin for 24 hr. Flow cytometry analysis was conducted to determine the rate of cell apoptosis with the FITC Annexin V Apoptosis Detection Kit I (Becton‐Dickinson; Brea, CA) following the manufacturer's instructions.

### 2.8 Evans blue extravasation

The content of Evans Blue (Sigma Aldrich) extravasation in the brain was measured to evaluate BBB permeability 3 days after ICH as previously (Xu et al., 2017). Mice were perfused with phosphate buffered saline (PBS) and sacrificed 2 hr after Evans Blue dye (2%; 4 ml/kg) was given intravenously. Brain hemispheres were collected and weighed immediately, and then homogenized in 1 ml lysis buffer containing 50% trichloroacetic acid. The homogenates were centrifuged at 12,000 *g* for 20 min and the absorbance of the supernatant was determined at 620 nm using a spectrophotometer (BioTek, Winooski, VT). A standard curve of the dye was prepared to calculate micrograms dye per gram of brain tissue.

### 2.9 Immunostaining

A double immunofluorescene procedure using NLRP3 (1:100, Santa Cruz Biotechnology; Santa Cruz, CA), CD31 (BD Biosciences; San Diego, CA), MAP2 (1:100, Merck/Millipore; Jaffrey, NH), GFAP (1:100, Merck/Millipore), and Iba‐1 (1:100, Abcam; Cambridge, MA) was performed as described previously. Photographs were taken with a confocal microscope (Leica; Solms, Germany) for further analysis.

### 2.10 Real‐time polymerase chain reaction (PCR) analysis

Total RNA from the ipsilateral hemispheres was extracted with Trizol Reagent (TaKaRa Bio; Shiga, Japan) and subsequently used for reverse transcribtase (RT) to generate cDNA using a PrimeScript RT reagent kit (TaKaRa Bio). Quantitative real‐time PCR (qPCR) was performed on all samples using the following primers: IL‐18 (sense 5′‐CCTACTTCAGCATCCTCTACTGG‐3′ and antisense 5′‐AGGGTTTCTTGAGAAGGGGAC‐3′); IL‐1β (sense 5′‐GCAACTGTTCCTGAACTCAACT‐3′ and antisense 5′‐ATCTTTTGGGGCGTCAACT‐3′); tumor necrosis factor (TNF)‐α (sense 5′‐CCCTCACACTCAGATCATCTTCT‐3′ and antisense 5′‐GCTACGACGTGGGCTACAG‐3′); NLRP3 (sense 5′‐ATTACCCGCCCGAGAAAGG‐3′ and antisense 5′‐TCGCAGCAAAGATCCACACAG‐3′). The primers were constructed by Invitrogen Corp. (Carlsbad, CA). The relative levels of gene expression were analyzed using SDS software (Applied Biosystems; Foster City, CA), and the results are expressed as fold differences.

### 2.11 Western blot analysis

For the Western blot analyses, the samples from perihematoma region of striatum were lysed in radioimmunoprecipitation assay buffer supplemented with Protease and Phosphatase Inhibitor Cocktail (Millipore; Bedford, MA). The primary antibodies used in the present study were as follows: ZO‐1 antibodies (Catalog NO. 61‐7300, 1:500 dilution, ThermoFisher), and NLRP3/ASC/Caspase‐1 antibodies (Catalog NO. sc‐66846/sc‐22514‐R/sc‐56036, 1:500 dilution Santa Cruz Biotechnology, CA). Image J software was used for intensity analysis.

### 2.12 Statistical analysis

Power analysis was performed by PASS 11(PASS software, Kaysville, Utah, USA). Alpha was set to be 0.05, and “1‐beta” was set to be 0.8. Quantitative data are presented as means ± *SD*. Data analyses were performed using *t* tests with the SPSS 20.0 program (SPSS Inc., Chicago, IL)., and all *p* < 0.05 were considered statistically significant. Graph presentation of the data was performed using GraphPad Prism 6 (GraphPad Software; San Diego, CA).

## 3 RESULTS

### 3.1 Glibenclamide alleviates cerebral edema, disrupted BBB, and neurological deficit after ICH

**Figure 1:**
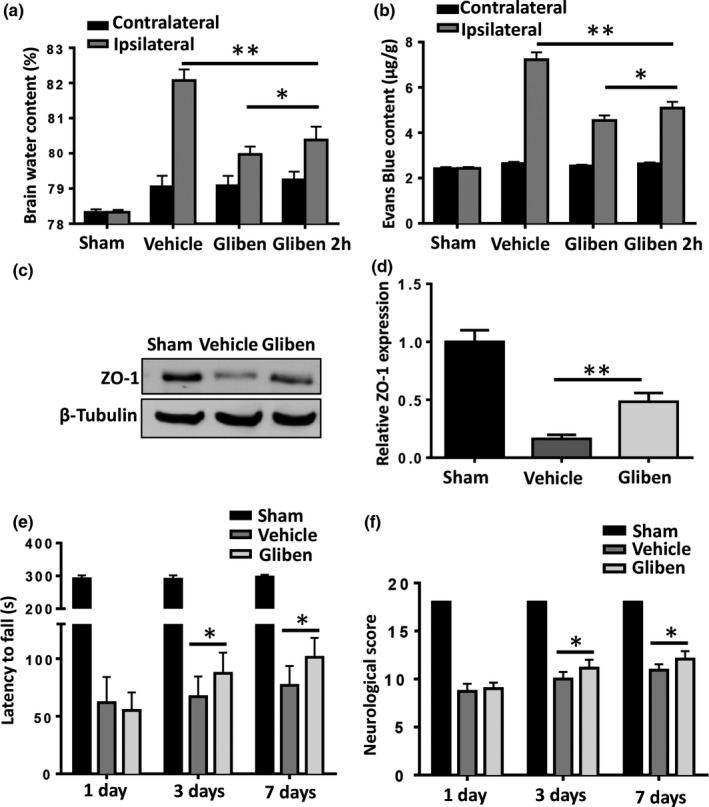
Glibenclamide attenuates brain edema and improves neurological outcomes in mice after intracerebral hemorrhage (ICH) by maintaining the integrity of the blood–brain barrier (BBB). (a) Compared to vehicle, glibenclamide significantly reduced the brain water content in the affected hemisphere after ICH. (b) Glibenclamide decreased the extravasation of Evans Blue in the brain after ICH. (c) Glibenclamide preserved the ICH‐induced degradation of the tight junction protein zona occludens‐1 (ZO‐1). (d) Glibenclamide improved neurological outcomes 3 and 7 days following ICH. (*n* = 4 in sham group, *n* = 6 in ICH group, *p < 0.05, **p < 0.01)

Brain water content increased in the ipsilateral hemisphere after ICH (82.1 ± 0.6%) compared with the sham group (78.3%, *p* < 0.01 vs. ICH). Treatment with glibenclamide significantly decreased edema (80.0%, *p* < 0.01 vs. ICH) 3 days after ICH (Figure 1a). BBB permeability after ICH was evaluated by Evans Blue (EB) extravasation. A significant increase in EB content was observed in the ipsilateral hemisphere 3 days after ICH compared with sham animals (Figure 1b, *p* < 0.01). Glibenclamide markedly reduced the extravasation of EB in the ipsilateral hemispheres of treated animals compared with the vehicle controls (Figure 1b, *p* < 0.01). Furthermore, Glibenclamide can still reduce brain edema and BBB disruption when administrated 2 hr after ICH (Figure 1a,b, *p* < 0.01). A western blot analysis was performed to evaluate the degradation of tight junctions; glibenclamide significantly inhibited the loss of ZO‐1 3 days after ICH (Figure 1c,d, *p* < 0.01). Garcia score and rotarod test were used to assess neurologic outcomes after ICH. Consistent with alleviating the edema and preserving the BBB, glibenclamide significantly improved neurological function 3 days post‐ICH compared with vehicle mice (Figure 1e,f, *p* < 0.05 at 3 and *p* < 0.05 at 7 days, respectively)

### 3.2 Glibenclamide reduces ICH injury‐induced cytokine production

**Figure 2:**
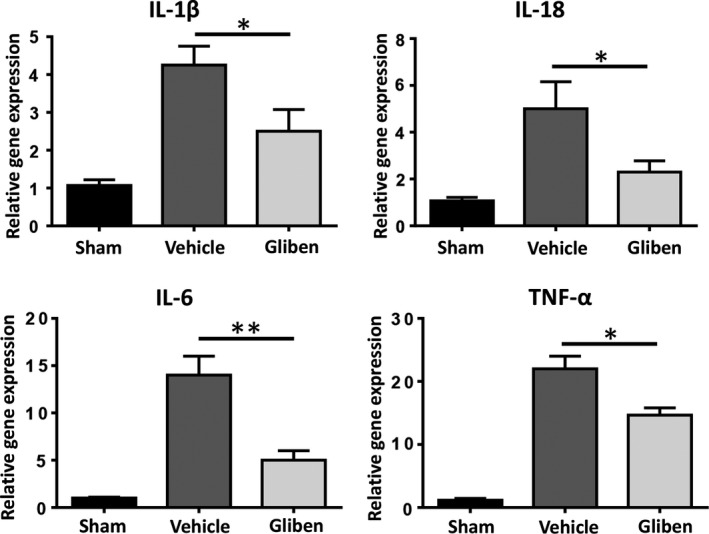
Glibenclamide decreases intracerebral hemorrhage (ICH)‐induced cytokine amplification. mRNA levels of interleukin (IL)‐1β, IL‐18, IL‐6, and tumor necrosis factor (TNF)‐α in brain tissue 3 days after ICH (*n* = 4 in sham group, *n* = 6 in ICH group, *p < 0.05, ** *p* < 0.01).

The graph shows the relative mRNA levels of inflammatory cytokines including IL‐1β, IL‐18, IL‐6, and TNF‐α in the perihematoma regions increased markedly 3 days after ICH, compared with those in the sham group. Glibenclamide inhibited the production of IL‐1β, IL‐18, IL‐6, and TNF‐α (Figure 2, *p* < 0.05).

### 3.3 NLRP3 is upregulated following ICH

**Figure 3:**
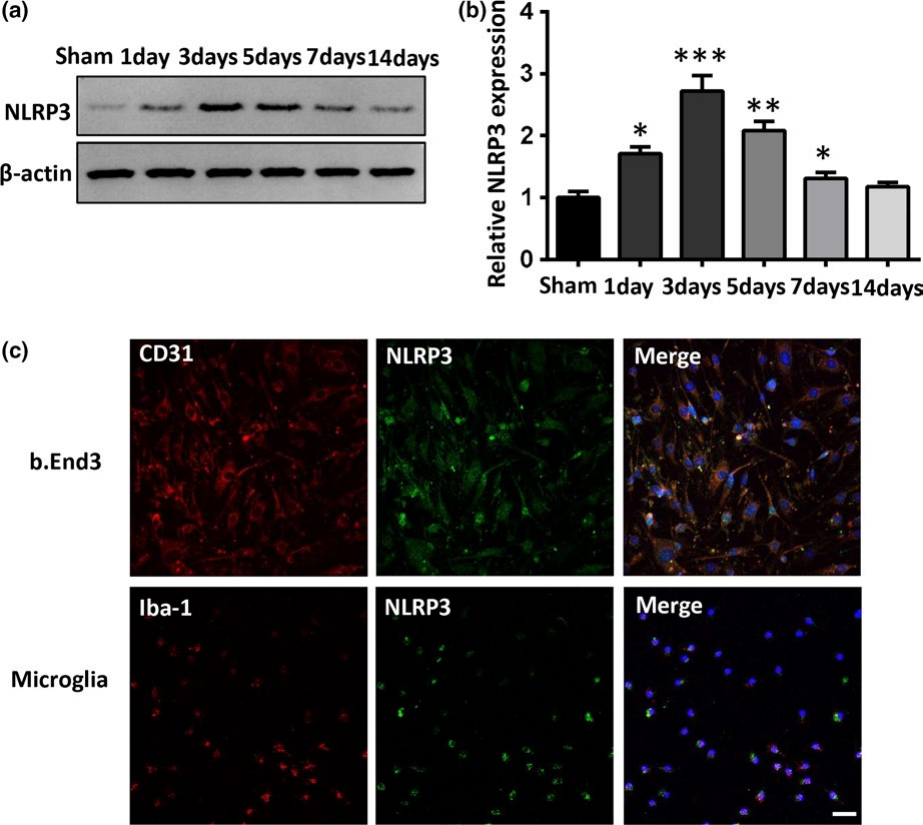
Changes in the nucleotide‐binding oligomerization domain‐like receptor with a pyrin domain 3 (NLRP3) protein after intracerebral hemorrhage (ICH). (a, b) Time‐course of NLRP3 protein expression in the perihematoma tissue. The expression of NLRP3 increased at 1 day, and reached a plateau at 3 days following ICH compared to the sham group (*n* = 3 in each group; compared to sham, *p < 0.05, **p < 0.01). (c) Representative NLRP3/CD31 or NLRP3/Iba‐1 immunofluorescent staining in b.End3 or primary microglia cells (scale bar = 100 μm).

Compared with the sham‐operated animals, the level of NLRP3 in mouse brain significantly increased starting at 1 day, reached a peak at 3 days, decreased but still elevated at 7 days, and nearly unchanged at 14 days after ICH (Figure 3a,b). The cellular expression of NLRP3 in the brain was examined in isolated mouse cells using GFAP/NLRP3, NeuN/NLRP3, Iba‐1/NLRP3, and GFAP/CD31 double immunolabeling procedure. NLRP3 was particularly expressed in the cytosol of microglia and microvascular endothelial cells (Figure 3c).

### 3.4 Glibenclamide reduces hemin‐induced cerebral microvascular endothelial cell death

**Figure 4:**
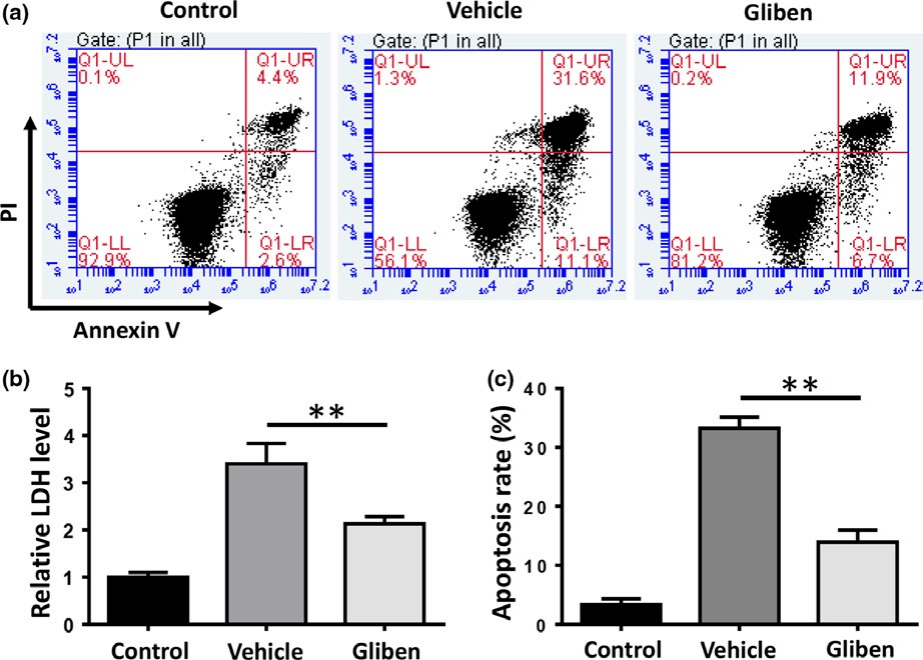
Glibenclamide reduces hemin‐induced cellular toxicity. (a) Flow cytometry analysis of cell apoptosis of b.End3 cells exposed to 60 μM hemin. (c) The apoptosis rate was plotted in a histogram. (b) Cell death was evaluated by the lactated dehydrogenase (LDH) release assay. (*n* = 3, *p < 0.05, **p < 0.01)

Necrosis of bEnd.3 endothelial cells was assessed by extracellular LDH assay 24 hr after the hemin treatment. As shown in Figure 1, exposing the bEnd.3 cells to 60 μM hemin led to a three‐ to‐ fourfold increase in the extracellular LDH level. Glibenclamide significant decreased extracellular LDH levels in bEnd.3 endothelial cells (Figure 4b, *p* < 0.01). Cell apoptosis was analyzed by flow cytometry‐based assays. The results showed that glibenclamide markedly decreased the proportion of cells undergoing apoptosis (Figure 4a,c, *p* < 0.01). These findings suggest that glibenclamide protects microvascular endothelial cells from death and glibenclamide serves as a BBB modulator.

### 3.5 Glibenclamide inhibits hemin‐induced NLRP3 activation in bEnd.3 cells

**Figure 5:**
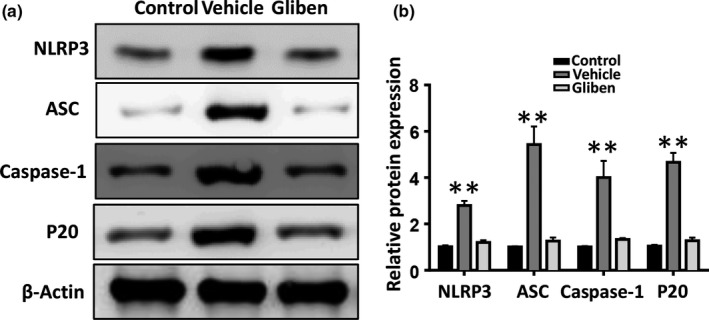
Glibenclamide inhibits the activation of hemin‐induced nucleotide‐binding oligomerization domain‐like receptor with a pyrin domain 3 (NLRP3) inflammasome. (a) Western blot analyses show a significant decrease in NLRP3, ASC, caspase‐1, and cl‐caspase‐1 (P20) protein expression in b.End3 cells 24 hr after the hemin treatment. (b) Bar graphs summarize the data, presented as means ± *SE*, from three groups (*n* = 3, *p < 0.05, **p < 0.01)

We evaluated the protein expression of the NLRP3 inflammasome contents, including NLRP3, ASC, caspase‐1, and cl‐caspase‐1 (P20) in bEnd.3 cells 24 hr after the hemin treatment. A western blot analysis revealed that hemin significantly upregulated NLRP3, ASC, caspase‐1, and cl‐caspase‐1 (P20) expression, indicating that hemin‐induced NLRP3 inflammasome activation in bEnd.3 cells. Glibenclamide significantly reduced hemin‐induced upregulation of NLRP3, caspase‐1, and ASC (Figure 5a,b, *p* < 0.01). These results demonstrate that glibenclamide effectively inhibits the activation of NLRP3 inflammasome components in microvascular endothelial cells after ICH.

### 3.6 NLRP3 knockdown reduces cell death and loss of ZO‐1 in bEnd.3 cells

**Figure 6:**
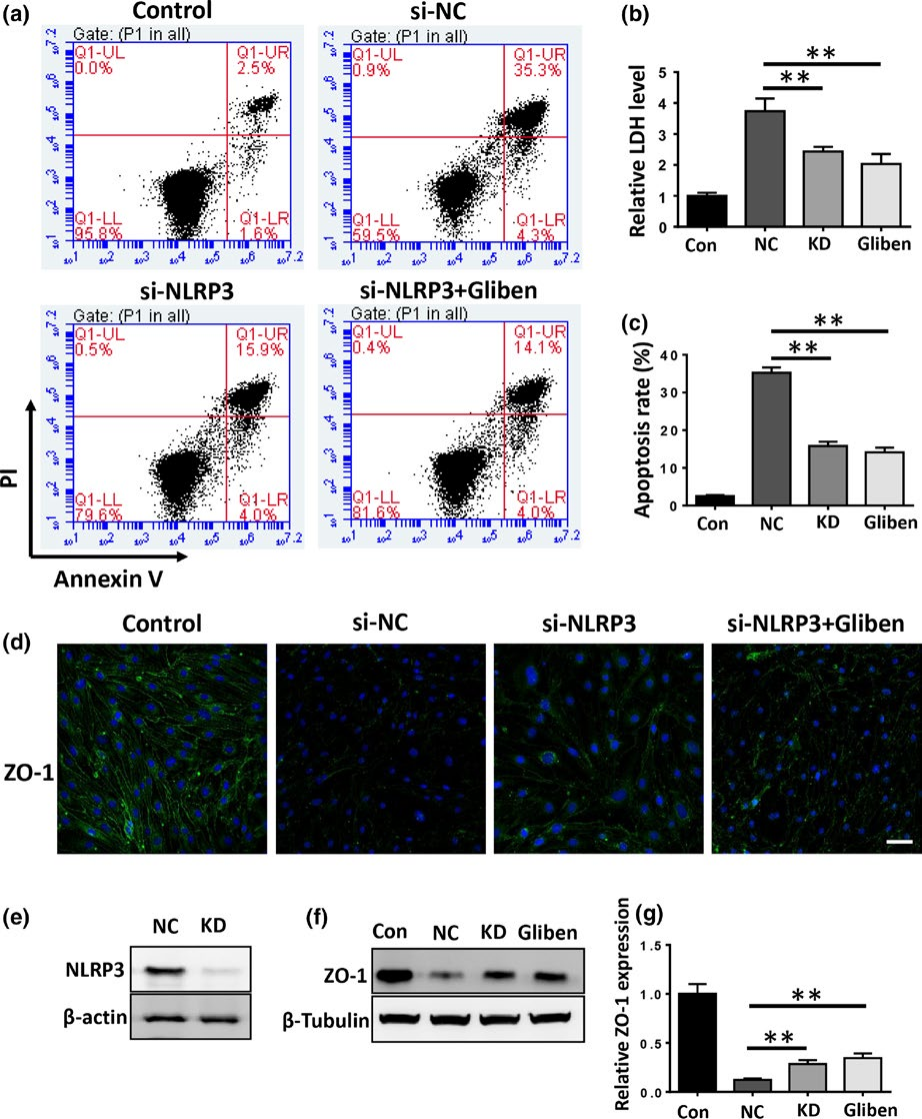
Nucleotide‐binding oligomerization domain‐like receptor with a pyrin domain 3 (NLRP3) knockdown reduces hemin‐induced cellular toxicity and degradation of zona occludens‐1 (ZO‐1), abolishing the protective effect of glibenclamide. (a) Flow cytometry analysis of cell apoptosis in the control, si‐NC, and si‐NLRP3 transfected b.End3 cells treated with or without glibenclamide. (b) Cell death was evaluated by the LDH release assay. (c) The apoptosis rate was plotted as a histogram. (d) Western blot analyses show that NLRP3 knockdown significantly increased hemin‐induced ZO‐1 degradation. (*n* = 3, *p < 0.05, **p < 0.01)

To further investigate the effects of NLRP3 on endothelial cell death and disruption of the BBB, siRNAs against NLRP3 were transfected into bEnd.3 cells. Knockdown efficiency was confirmed by western blot analysis (Figure 6e). Our results showed that knockdown of NLRP3 reduced the extracellular LDH level (Figure 6b, *p* < 0.01), cell apoptosis (Figure 6a,c, *p* < 0.01), and loss of ZO‐1 (Figure 6d,f,g, *p* < 0.01) when compared to the scrambled siRNA control group. However, NLRP3 knockdown abolished the protective effect of glibenclamide.

## 4 DISCUSSION

Growing evidence suggests that glibenclamide inhibits neuroinflammation and improves behavioral outcomes following CNS injury (Zhang et al., 2017). Our present results demonstrate that glibenclamide attenuated cerebral edema, neuroinflammation, disruption of the BBB, and neurological deficit after ICH. Additionally, glibenclamide reduced hemin‐induced cell death and loss of ZO‐1 in bEnd.3 cells. These effects were probably due to the regulation of the NLRP3 inflammasome activation. Our findings demonstrate that glibenclamide exerted neuroprotective effects after ICH, possibly by inhibiting the activation of NLRP3 inflammasome.

In addition to its hypoglycemic effects, glibenclamide has been shown to play a protective role in inflammation‐related disorders, particularly in CNS injury, including traumatic brain injury (Patel, Gerzanich, Geng, & Simard, 2010; Xu et al., 2017), ischemia‐reperfusion injury (Abdallah, Nassar, & Abd‐El‐Salam, 2011; Caffes et al., 2015; Gao et al., 2016; Yang et al., 2014), subarachnoid hemorrhage (SAH) (Simard et al., 2009; Tosun et al., 2013), and ICH (Jiang et al., 2017; Ma et al., 2014). Glibenclamide significantly attenuates neuroinflammation, decreases disruption of the BBB, and improves memory function by blocking the Sur1–Trpm4 channel in both experimental SAH and ICH (Zhou, Shi, Wang, Chen, & Zhang, 2016). Another study suggested that inhibiting microglial Sur1‐Trpm4 channels downregulates the transcription of inducible nitric oxide synthase, leading to reduced activation of microglia and an inflammatory response in the CNS (Kurland et al., 2016; Tosun et al., 2013). Similarly, glibenclamide significantly reduces cerebral edema, infarct volume, production of inflammatory mediators, and neutrophil infiltration in the ischemic brain by inhibiting Sur1 and the regulatory subunit of the K_ATP_ channel (Abdallah et al., 2011; Ortega et al., 2012). Glibenclamide has been reported to reduce brain edema and endothelial apoptosis by inhibiting activity of the JNK/c‐jun pathway after traumatic brain injury (Xu et al., 2017). Consistent with previous studies, we showed here that glibenclamide significantly attenuated brain edema and neurological deficit after ICH. Our data indicate that glibenclamide is a potent therapeutic strategy for ICH.

The sterile inflammatory reaction is important in the pathophysiology of ICH (Zhou et al., 2016). Thrombin and hemoglobin breakdown products after ICH can trigger a sterile inflammatory response through the NLRP3 inflammasome. Genetic depletion of NLRP3 significantly attenuates inflammation and injury after ICH, indicating that drugs that can manipulate the activation of NLRP3 inflammasome are promising therapeutic strategies for patients with ICH (Yuan et al., 2015). Several in vivo and in vitro experiments have confirmed that glibenclamide can effectively inhibit the activation of NLRP3 inflammasome Zhang et al., 2017). A plausible mechanism underlying inhibition of the NLRP3 inflammasome is that glibenclamide prevents cellular efflux of K^+^ by inhibiting the regulatory subunit of ATP‐sensitive potassium channels (K_ATP_) on the cell membrane. It is known that an intracellular drop in K^+^ (<70 mM) induces the activation of NLRP3 inflammasome (Lamkanfi et al., 2009). Inappropriate activation of the NLRP3 inflammasome leads to activation of caspase‐1 and production of the proinflammatory cytokines IL‐1β and IL‐18, which exacerbate inflammation and tissue damage (Zhou et al., 2016). Glibenclamide inhibits the activation of NLRP3 inflammasome and protects against inflammation and tissue damage in many inflammatory diseases, including bronchopulmonary dysplasia (Liao et al., 2015), allergic asthma (Cui et al., 2015), acute pancreatitis (York, Castellanos, Cabay, & Fantuzzi, 2014), sepsis (Koh et al., 2011), and atherosclerosis (Ling et al., 2013). In the CNS, glibenclamide ameliorates SAH‐induced BBB permeability, proinflammatory cytokine expression, and neuronal cell death by inhibiting activation of NLRP3 (Simard et al., 2009). Glibenclamide also decreases ischemic brain injury‐induced TNF‐α and prostaglandin E2 expression, infiltration of neutrophils, and vascular permeability (Abdallah et al., 2011). In this study, glibenclamide significantly reduced ICH injury induced by IL‐1β, IL‐18, IL‐6, and TNF‐α production and disruption of the BBB. These results were consistent with previous research showing that glibenclamide treatment markedly protects against ICH‐induced BBB disruption and inflammatory response.

Although the anti‐inflammatory effects and mechanisms of glibenclamide have been well studied, the exact glibenclamide mechanism of action related to BBB integrity in the CNS is not clear. Previous studies have demonstrated that NLRP3 is mainly expressed in immune organs and immune cells, as well as in the CNS (Liu et al., 2013). We found here that NLRP3 was mainly expressed in the cytosol of microglia and endothelial cells but not in astrocytes and neurons and the expression of NLRP3 was significantly increased in the ipsilateral brain after ICH. The protective effects of glibenclamide on BBB integrity, together with the finding that the NLRP3 inflammasome was expressed in endothelial cells after ICH, suggest that the benefit of glibenclamide involves its action on endothelial cells.

Cerebral microvessel endothelial cells have tight junction proteins that are essential for maintaining BBB integrity (Keep et al., 2008). Our results clearly demonstrate that glibenclamide reduced hemin‐induced endothelial cell death and that glibenclamide significantly decreased the NLRP3 inflammasome upregulation and subsequent ASC and caspase‐1 expression in bEnd.3 cells. These results indicate that glibenclamide protects the integrity of the BBB against ICH injury, probably by inhibiting the activation of NLRP3 inflammasome in microvascular endothelial cells. To evaluate this further, we knocked down bEnd.3 NLRP3 expression with siRNAs and observed that depletion of NLRP3 reduced hemin‐induced cell death and loss of ZO‐1 compared to the scrambled siRNA control group. However, knockdown of NLRP3 abolished these protective effects of glibenclamide. Our in vivo results also show that glibenclamide significantly inhibited loss of ZO‐1 and reduced the disruption of BBB 3 days after ICH. Taken together, these results suggest for the first time that glibenclamide maintained BBB integrity by inhibiting the activation of NLRP3 inflammasome and subsequent endothelial cell death and loss of tight junction proteins after ICH.

The BBB is mainly formed by cerebral endothelial cells, and their linkage of tight junctions is essential for homeostasis in the CNS (Keep et al., 2008). ICH‐induced disruption of the BBB contributes to edema formation, increases in cytokines and chemokines, infiltration by leukocytes, and activation of matrix metalloproteinases in the perihematomal brain, causing a vicious cycle often culminating in further disruption of the BBB. Our data indicate that glibenclamide broke this cycle by protecting endothelial cells from ICH injury via NLRP3 signaling. A link has been reported between activation of inflammasomes and apoptosis. During programmed cell death, mitochondrial dysfunction triggers NLRP3‐dependent activation of the inflammasome, which can be inversely regulated by Bcl‐2 and 8‐hydroxy‐2'‐deoxyguanosine (Shimada et al., 2012). By inhibiting activation of NLRP3, we found here that glibenclamide significantly reduced hemin‐induced apoptosis of endothelial cells. The results of this study provide new evidence that inhibiting the NLRP3 inflammasome with glibenclamide offers the benefit of maintaining BBB integrity and reducing ICH injury.

In conclusion, we demonstrated that glibenclamide maintains the integrity of the BBB in experimental ICH and does so, in part, by inhibiting the activation of NLRP3 inflammasome in microvessel endothelial cells. Our findings may contribute to the further elucidation of the pharmacological mechanism of action of glibenclamide and assist in the development of a novel therapeutic strategy for treating clinical ICH.
